# Wafer-Scale Electrical Characterization of Al/Al_*x*_O_*y*_/Al Tunnel Junctions for Process Monitoring at Room Temperature

**DOI:** 10.3390/nano16100569

**Published:** 2026-05-07

**Authors:** Simon Johann Klaus Lang, Ignaz Eisele, Johannes Weber, Alexandra Schewski, Emir Music, Alwin Maiwald, Martin Hahn, Daniela Zahn, Zhen Luo, Lars Nebrich, Benedikt Schoof, Thomas Mayer, Leonhard Sturm-Rogon, Wilfried Lerch, Rui Nuno Pereira, Christoph Kutter

**Affiliations:** 1 Fraunhofer Institute for Electronic Microsystems and Solid State Technologies EMFT, 80686 Munich, Germany; ignaz.eisele@emft.fraunhofer.de (I.E.); johannes.weber@emft.fraunhofer.de (J.W.); alexandra.schewski@emft.fraunhofer.de (A.S.); emir.music@emft.fraunhofer.de (E.M.); alwin.maiwald@emft.fraunhofer.de (A.M.); martin.hahn@emft.fraunhofer.de (M.H.); daniela.zahn@emft.fraunhofer.de (D.Z.); lars.nebrich@emft.fraunhofer.de (L.N.); thomas.mayer@emft.fraunhofer.de (T.M.); leonhard.sturm-rogon@emft.fraunhofer.de (L.S.-R.); wilfried.lerch@emft.fraunhofer.de (W.L.); rui.pereira@emft.fraunhofer.de (R.N.P.); 2 Center Integrated Sensor Systems (SENS), Universität der Bundeswehr München, 85577 Munich, Germany; 3 School of Computation, Information and Technology (CIT), Technical University of Munich, 85748 Garching, Germany; zhen.luo@tum.de (Z.L.); benedikt.schoof@tum.de (B.S.)

**Keywords:** tunnel junctions, wafer-scale process control monitoring, advanced electrical characterization

## Abstract

Josephson junctions are key elements in superconducting qubits. Their efficient wafer-scale characterization is crucial for process control and optimization, motivating analysis approaches that extend beyond conventional cryogenic measurements. In this work, we demonstrate that room temperature (RT) capacitance and current–voltage measurements, combined with appropriate data analysis, enable extraction of relevant junction parameters such as oxide thickness, tunnel coefficient, and interfacial defect density. Furthermore, different charge transport mechanisms can be identified from detailed current–voltage analysis. We evaluate our characterization technique using tunnel junctions fabricated on 200 mm wafers in a complementary metal–oxide–semiconductor (CMOS)-compatible subtractive process. The results show a homogeneous average oxide thickness across the wafer with a variation below 3%. A dependence of the tunnel coefficient on oxide thickness indicates a stoichiometry gradient within the oxide. Additionally, low interfacial defect densities in the range of 70–5000 defects/cm^2^ are observed in our junctions, increasing with decreasing oxide thickness, suggesting that wet etching used for thickness control introduces interfacial trap states. Our study highlights the importance of advanced RT characterization for extracting tunnel junction parameters on the wafer scale, enabling effective process monitoring and optimization in industrial superconducting qubit manufacturing.

## 1. Introduction

Josephson junctions, i.e., tunnel junctions (TJs) that enable the Josephson effect, are a key element of many different devices, such as traveling wave parametric amplifiers, superconducting quantum interference devices and superconducting qubits, which operate at cryogenic temperatures. The development of time-efficient and wafer-scale characterization of TJs, which can be also used for process control monitoring (PCM), is currently a major research topic. An efficient approach should involve primarily room-temperature (RT) electrical measurements since cryogenic characterization of TJs is too time-consuming and unsuitable for wafer-scale characterization, especially for PCM, which relies on fast feedback loops. Additionally, a complementary metal–oxide–semiconductor (CMOS)-based fabrication environment is essential for scalability and enhanced precision towards industrialization and commercialization of JJ devices.

A recent study [1] showed that with industry-compatible fabrication of Al/${\mathrm{Al}}_{x}O_{y}$/Al TJs, transmon-style qubits with a high yield of 98.5% and energy relaxation times ($T_{1}$) of up to 167 µs could be fabricated, approaching values typically achieved with conventional laboratory-style fabrication based on double angle evaporation techniques [2,3,4]. Here, the authors used RT wafer-scale resistance measurements of their TJs to study the frequency variability of their qubit devices. It was found that junction resistance targeting is limited by variations in the TJs’ oxide barrier height instead of the junction area. This suggested that oxide thickness and barrier height are critical parameters, but which of the two dominates and how their variability can be characterized and controlled during fabrication remain open. A deeper understanding of such questions requires thorough wafer-scale statistical analysis of TJs, with parameters varied in a well-defined manner.

In the present study, we show that essential tunnel junction parameters (e.g., oxide thickness, effective barrier height, and interfacial defect densities) can be obtained on the wafer scale from advanced electrical characterization and proper data analysis. Based on the detailed study of current vs. voltage characteristics, different charge transport mechanisms across the junctions can also be distinguished. These analytical methods are applied to study tunnel junctions fabricated on 200 mm wafers with industrially scalable subtractive processing based on CMOS tools similar to that reported in recent studies [1,5]. The analysis provides insights into oxide thickness values and wafer-scale homogeneity, oxide barrier height and related stoichiometry gradients, as well as into interfacial defect densities. Our study exemplifies the importance of the proposed advanced electrical characterization principles for studying TJs and their wafer-scale parameter distributions, which is essential for proper process control and optimization in an industrial environment.

## 2. Experimental

**Figure 1:**
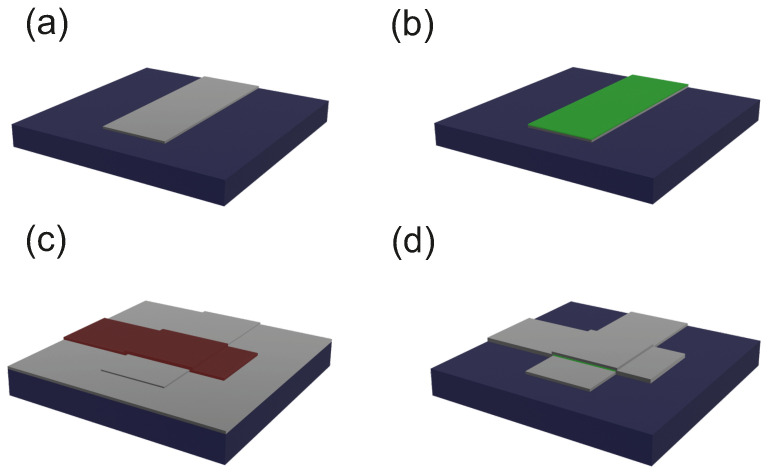
Scheme of the fabrication process of the TJs in the CMOS pilot line: (**a**) bottom Al (grey) electrode after structuring on silicon (blue), (**b**) wet-chemical oxide etching (green), (**c**) top Al deposition and lithography of second layer, (**d**) structuring of top electrode.

The fabrication process of our CMOS-compatible Al/${\mathrm{Al}}_{x}O_{y}$/Al TJs is quite similar to a previously reported process [6]. However, a few variations were implemented, as described below, to enable a wider range of oxide thicknesses, required for the present study. The substrates consisted of high-ohmic (100) p-type 200 mm silicon wafers with a resistivity of 3–5 kΩcm. Prior to deposition of the first Al layer, an ex situ liquid HF dip for 3 min was performed for removal of the native silicon oxide. In a cluster-tool (Clusterline 200, Evatec AG, Trübbach, CHE), after an initial pre-heating step, 120 nm of Al was deposited. The bottom electrode layer was lithographically patterned using an i-line stepper (Canon). A dry etch step was performed in an AMAT P5000 system (Applied Materials, Santa Clara, CA, USA) using a chlorine-based etch gas. We note that during the photoresist strip, which was conducted using H_2_O plasma in the same device as the dry etching, the surface of the bottom Al electrode became further oxidized beyond the native aluminum oxide that results from vacuum breakage during lithography (Figure 1a). The thickness of the Al surface oxide resulting from the H_2_O plasma was measured with ellipsometry to be around 4.4 nm. Ellipsometry is an optical method used to study the dielectric properties of thin films by analyzing the change in the polarization of light due to reflection or transmission, and these changes are compared to a theoretical model. This technique has become the standard for determining the thickness of silicon oxide layers in the semiconductor industry.

We actually used this oxide as a reference ${\mathrm{Al}}_{x}O_{y}$ layer in the TJs. To achieve oxides with different thicknesses, we performed subsequent wet etching in phosphoric acid for different durations (10 s, 20 s, 30 s) (Figure 1b) to manufacture our samples (etch10, etch20 and etch30; see Table 1). For the reference sample, no wet etching of the H_2_O plasma oxide was performed. The etching process was followed by the deposition of the top electrode. It consists of 100 nm Al deposited in the cluster-tool similar to the bottom layer to complete the JJ (Figure 1c). During the etching of the top layer (Figure 1d), precautions were taken to avoid etching through the bottom Al electrode as the process lacks selectivity.

**Figure 2:**
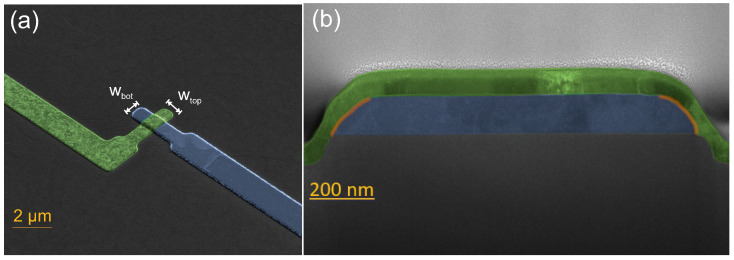
(**a**) Top view of a 1 µm × 1 µm JJ with top (green) and bottom (blue) electrodes. (**b**) TEM cross-section of the same JJ with orange-marked sidewall region of the junction overlap area.

To analyze the leakage current of the TJs, test structures with different sizes, e.g., varying widths of the top ($w_{top}$) and bottom ($w_{bot}$) electrodes, were designed (Figure 2a). The resulting overlap area of a JJ can be simplified to the top area, defined as $A=w_{top}\cdot w_{bot}$. Later on, the sidewall area (orange region, Figure 2b), defined as $A_{S}=2\cdot h\cdot w_{top}$, where *h* is the thickness of the bottom Al layers, will be taken into account in the detailed analysis of the current flow across the junction. Comparing the current flow for different samples with varying geometries enables a comprehensive oxide analysis. In total, 256 different test structures with *A* ranging from 0.12 µm^2^ to 1600 µm^2^ were included in each single chip.

The electrical measurements were conducted in a fully automated wafer prober, which could handle an entire box of wafers. The layout for the stepping could be directly extracted from the lithography process. It is important to emphasize that without the calibration test structures that included all the metal lines except for the junction crossing, it would have been impossible to achieve high measurement accuracy in the capacitance measurements down to fF. These structures are essential for enabling the effective assessment of even very small junction areas (∼5 µm^2^), which would otherwise remain undetectable. The two-point resistance measurement had an accuracy limited by the contact resistance, on the order of Ω, which is negligible in the context of MΩ junction resistance.

For the reference wafer, thin lamellae (∼50 nm) were prepared from the finalized TJs located at the wafer center with focused ion beam in a SEM (Thermo Fisher, Waltham, MA, USA) (scanning electron microscope) for the structural analysis of the oxide thickness $t_{ox}^{EELS}$ by the oxygen concentration via TEM (transmission electron microscopy) and EELS (electron energy loss spectroscopy) measurements (provided by SGS Fresenius Institut, Dresden, GER). The EELS technique measures the energy loss experienced by electrons as they interact with atoms while traversing a thin lamella. In our case, this method enables the identification of the oxygen position and content in the TJs. TEM cross-section images are formed by the interaction of the electron beam with the transmitted lamella. As high-energy electrons have a minor wavelength compared to optical light, even features down to nm sizes are identifiable with this method.

## 3. Results and Discussion

### 3.1. Wafer-Scale Capacitance Measurements

Wafer-scale capacitance measurements were conducted for junctions with varying overlap areas *A* between 1 µm^2^ and 1600 µm^2^. In detail, a mean capacitance $\bar{C}$ was calculated from across-wafer capacitance measurements of a specific A. The relative standard deviation (RSD) of the capacitance was below $RSD_{\bar{C}}<3$% for all samples.

**Figure 3:**
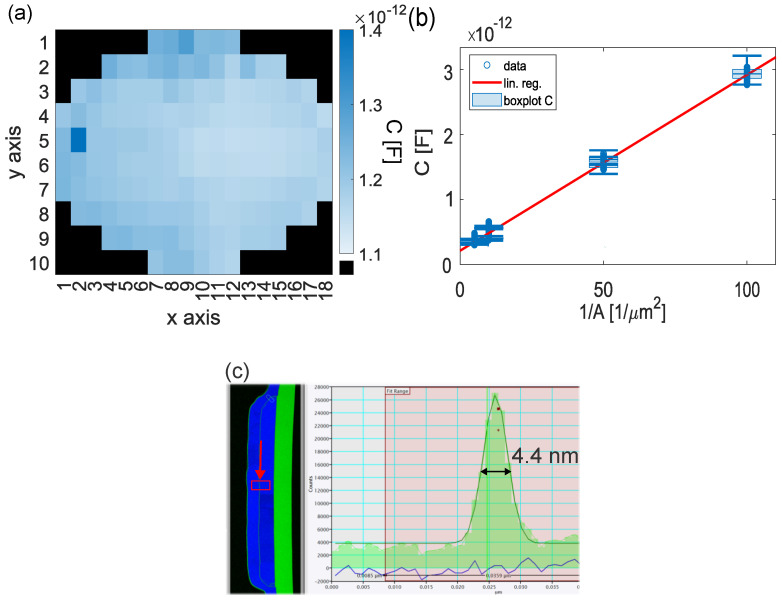
(**a**) Across-wafer capacitance map with 140 junctions of area *A* = 50 µm^2^ with $\bar{C}=1.19$ pF, relative standard deviation $RSD_{\bar{C}}=2.3$% and functional devices ($10^{-14}$ < C < $10^{-8}$) of 98.6%. (**b**) Capacitance *C* vs. area *A* for the reference wafer with a linear regression (the capacitances of the single TJs are indicated with circles). (**c**) Oxygen distribution measured with EELS of a junction for the reference sample. Measurement spot indicated with arrow (red) between the top and bottom aluminum electrodes (blue). FWHM of the Gaussian oxygen peak was measured to be $t_{ox}^{EELS}=4.4$ nm.

An example of a capacitance map is shown in Figure 3a. By using multiple overlap areas and applying a simple plate capacitor model

(1) $$C/A=\frac{\varepsilon_{r}\varepsilon_{0}}{t_{ox}}$$

the ratio C/A can be obtained analogously for all samples (see Table 1) by a linear regression with a crossing with the y-axis close to the origin; i.e., design and fabricated areas agree with a minor offset. An example is shown for the etch20 sample in Figure 3b.

The dielectric constant $\varepsilon_{r}$ can be calculated directly from the *C*/*A*, with $\varepsilon_{0}$ being the vacuum permittivity, if the oxide thickness $t_{ox}$ is known. Therefore, EELS measurements on a single junction of the reference wafer’s center were performed. By analyzing the FWHM of the oxygen Gaussian distribution, $t_{ox}^{EELS}=4.4$ nm (Figure 3c) was calculated, which is in agreement with ellipsometry measurements (see Section 2). For the reference TJs, this resulted in $\varepsilon_{r}$∼10, which is higher than the commonly known value of $\varepsilon_{r}$∼8, but still in agreement with other investigations [7]. Assuming that $\varepsilon_{r}$ remains approximately constant for all samples measured in this work, $t_{ox}$ for the etched samples could be calculated from the capacitance measurements using (1) (see Table 1). Variations in $\varepsilon_{r}$ arising from differences in stoichiometry are assumed to be minor for our RA range (see Table 1) [8] and therefore are neglected.

In this manner, ${\bar{t}}_{ox}$ is the across-wafer mean value with a relative standard deviation across the wafer of $RSD_{{\bar{t}}_{ox}}∼RSD_{\bar{C}}<3$%, defined by the capacitance measurement. This makes the capacitance measurements a highly accurate, time-efficient process-monitoring technique during wafer processing, not only for single junctions, but also for the entire wafer, compared to the known time-intensive single-die TEM measurements [9].

### 3.2. Current–Voltage (I-V) Characteristics

**Figure 4:**
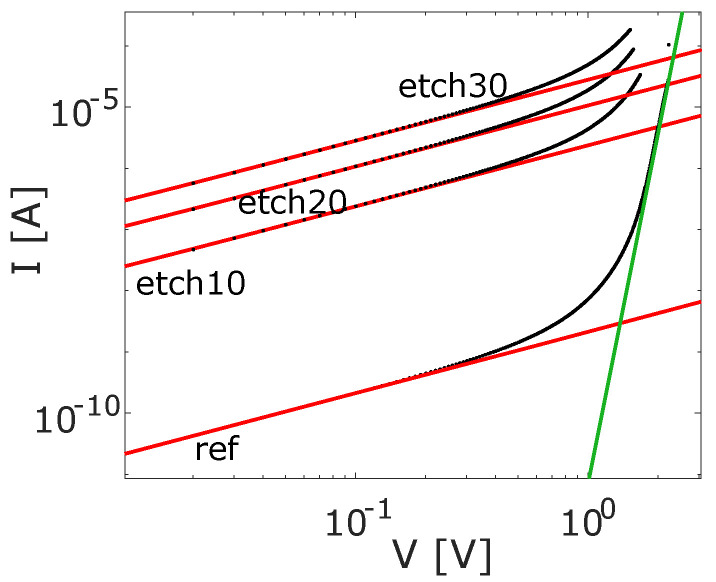
I-V curves of TJs with size *A* = 25 µm^2^ from the different wafers’ centers with fitted direct (red) and Fowler–Nordheim (green) tunneling.

In recent years, RT measurements [10] have become a common method to determine the approximate DC resistances (∼10 kΩ) [11,12] of Josephson junctions, especially in the framework of optimization [13,14]. Using the Ambegaokar–Baratoff relation [15], one can directly infer the critical current of the junction, which represents its Josephson energy. Since this value, along with the capacitive energy, determines the qubit frequency [1] (∼4.5 GHz) and the noise dependency of the qubit, it is a central parameter for the qubit characterization, in particular its frequency targeting. In our study, current–voltage IV curves were recorded to investigate the TJ’s tunnel transport in detail. The IV characteristics can be obtained by gradually increasing the voltage across the junction while measuring the corresponding current flow. Slow sweeping rates are important for high accuracy. As examples, the RT electrical data of the reference and the etched TJs are shown in Figure 4 (measurements taken from TJs in the center of the wafers).

An examination of the I-V curves indicates the presence of multiple tunneling mechanisms, making it essential to account for different tunneling regimes. At low voltage *V* (<0.3 V), direct tunneling (DT) appears for all samples (Figure 4, red line) as it is the case for trap-free insulators [10]

(2) $$I_{DT}=\frac{\alpha kAV}{t_{ox}}\exp\left(-kt_{ox}\right),$$

with $\alpha =\frac{e}{8\beta^{2}\pi^{2}\hbar }$, tunnel coefficient $k=2\beta \sqrt{\frac{2\Phi m^{\prime }}{\hbar^{2}}}$, average barrier height Φ of the oxide, effective electron mass m’, electron charge *e* and β, a correction factor for a non-rectangle shape of the potential barrier. With the wafer-mean oxide thickness ${\bar{t}}_{ox}$, *k* can be fitted for the different samples (Table 1). As *k* is dependent on both β and Φ, we can only estimate Φ for the reference sample. Here, EELS measurements (Figure 3c) revealed a Gaussian-shaped oxygen distribution across the junction oxide barrier. Therefore, we assume a comparable Gaussian shape of the potential barrier, which we calculate as β∼1, resulting in Φ∼3.14 eV for the reference sample using (2) with an effective electron mass of $m^{\prime }$ = 0.75 $m_{e}$. This value is in agreement with the literature [16,17].

This approach, however, only applies to Gaussian-shaped potential barriers, which may no longer be applicable to the etched samples. During the H_2_O plasma strip, we first perform a plasma densification of the oxide, which is modified after the wet etch. As the oxide begins to etch at the surface, the less densified (i.e., less stoichiometric) region is etched first, resulting in an overall increase in *k* (see Table 1). Given the non-homogeneous shape of the barrier, we should expect that both Φ and β change simultaneously, making it impossible to determine the individual impact of etching on each parameter.

Through further investigating the I-V curves, additional insights into traps in the oxide can be obtained. If traps are absent in oxides, at higher voltages, electrons will accumulate at the metal insulator interface as space charges and create an internal electric field. Thus, the current flow will be enhanced according to Mott–Gurney’s law [18]

(3) $$I_{MG}=\frac{9}{8}\frac{A\mu \varepsilon_{0}\varepsilon_{r}V^{2}}{t_{\mathrm{ox}}^{3}}\;$$

with the electron mobility μ. However, in real materials the presence of traps cannot be neglected. The so-called space charge limited current (SCLC) is defined by the initial filling of shallow traps with electrons (shallow trap-squared law), which can be described by multiplying (3) by a trap factor θ. Additional transport channels like (multi-step) trap-assisted tunneling (TAT) and Poole–Frenkel emission (PFE) will appear in parallel. PFE occurs due to trapped electrons in the insulator, which get thermally released by the conduction band bending caused by an applied voltage [19]. Additionally, traps in the oxide facilitate tunneling of electrons by reducing the tunnel distance. As single-trap-assisted tunneling events are improbable, a collective approach is commonly applied to take multiple intermediate tunneling processes into account. The modern SCLC (mSCLC), combining SCLC with PFE and TAT, describes the electron transport in oxides in a good approximation with a simple power law

(4) $$I_{mSCLC}∼V^{m}\;$$

where a power m>2 implies a deviation from the ideal Mott–Gurney law. For all our samples, this behavior cannot be observed (Figure 4), indicating the absence of traps in the oxide, prior to transitioning into the Fowler–Nordheim (FN) tunneling. This phenomenon is attributed to the reduction in the effective tunnel oxide width for electrons with the bending of the AlOx conduction band beneath the metal band edge, thereby enhancing current flow [20]

(5) $$I_{\mathrm{FN}}∼\frac{V^{2}}{\Phi t_{ox}^{2}}\exp\left(-\frac{bt_{ox}\Phi^{3/2}}{V}\right)$$

with the FN coefficient *b* = 6.83 eV^−3/2^ V/nm [21]. At elevated voltages (∼1 V), all samples exhibit the same gradient, which can be attributed to this tunneling process. In Figure 4, curve fitting (green) was attempted, which resulted in Φ = 1.04 eV, which is a factor of 3 off from literature values [16,17]. We conclude that the premature breakthrough prevents the accurate fitting of Φ from FN tunneling, as typically done.

### 3.3. Leakage Current Measurements

Resistance measurements were carried out on TJs with different overlap sizes across the central region of the wafers. For a more in-depth analysis, the simplified junction area $A=w_{top}$·$w_{bot}$ (top area) was extended with the sidewall area $A_{S}=2\cdot h\cdot w_{top}$, whereby *h* defines the height of the bottom Al layer, $w_{top}$ the width of the top electrode and $w_{bot}$ the width of the bottom electrode (see Section 2). This allows for the analysis of the location of leakage (sidewall) current. For the assumption of cross-wafer homogeneous oxide thickness and resistivity [22], the top area resistance (RA) and the sidewall area resistance ($RA_{S}$) for the junctions can be defined for the two regions, separately. For the junction resistance, this results in

(6) $$\frac{1}{R}=\frac{w_{top}\cdot w_{bot}}{RA}+\frac{2\cdot h\cdot w_{top}}{RA_{S}},$$

which transforms into

(7) $$R=\frac{RA\cdot RA_{S}}{w_{bot}\cdot RA_{S}+2\cdot h\cdot RA}\cdot \frac{1}{w_{top}}.$$

For the condition of $w_{bot}\cdot RA_{S}$>>2·h·RA, which is satisfied when $w_{bot}$ is sufficiently large, the equation can be further simplified to

(8) $$R=\frac{RA}{w_{top}\cdot w_{bot}}\;$$

**Figure 5:**
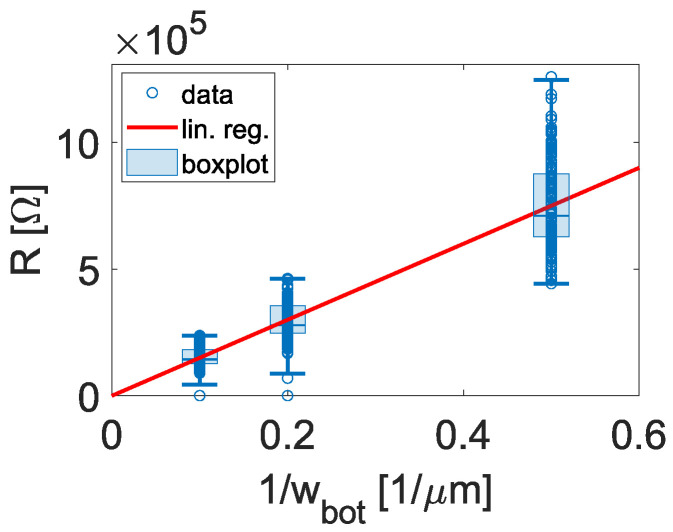
Cross-wafer JJ resistances potted against different bottom junction widths 1/$w_{bot}$. As an example, data of wafer etch30 is shown.

For the analysis of the region contribution to the resistance, (8) was used to fit RA while holding $w_{top}$ constant (see Figure 5, red line). This ensures that the condition for (8) is fulfilled, as $1/w_{bot}$ is further increased. Analogously to that, $RA_{S}$ is calculated by using RA and fitting the pre-factor in (7), while holding $w_{bot}$ constant. The results of RA and $RA_{S}$ (see Table 1) are quite similar for the same samples, indicating the absence of sidewall effects and a homogeneous flow of the current across the top and sidewall area. By comparing the different samples with each other, RA and $RA_{S}$ reduced with a decrease in the oxide thickness by prolonged oxide etching while maintaining comparability (Table 1). This behavior defines our etching as a homogeneous oxide reduction process.

### 3.4. Wafer-Scale Breakthrough Measurements

**Figure 6:**
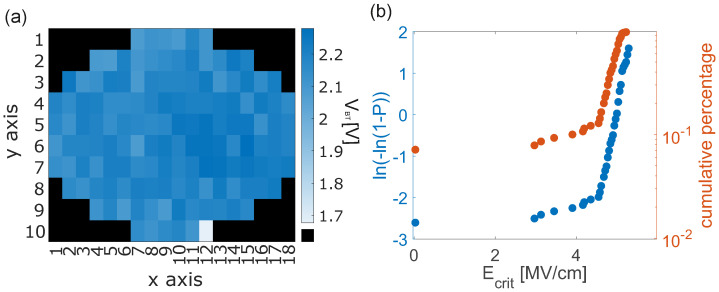
(**a**) Cross-wafer measurements of the breakthrough voltage for the reference sample. (**b**) ln{−ln(1−p)} (blue) and *p* (red) vs. *E* for the reference sample. At $E_{crit}$ = 4.6 MV/cm, the transition appears for $P_{k}$ = 12.9%.

In addition to resistance and capacitance measurements, the behavior of the junction can also be characterized by the breakthrough voltage $V_{BT}$, i.e., the voltage at which the junction undergoes electrical breakdown (shorting). For the measurements, the voltage was ramped up at a rate of 0.07 V/s in 10 mV increments. Wafer-scale measurements were performed on junction sizes of 5 × 5 µm^2^ (see Figure 6a). The measured across-wafer mean breakthrough voltage ${\bar{V}}_{BT}$ drops already from 2.18 V to 1.66 V (Table 1) after applying 10 s oxide etch. For additional etching times, only a minor decrease in ${\bar{V}}_{BT}$ is observable. The relative standard deviation of the breakthrough mean was $RSD_{{\bar{V}}_{BT}}\leq 4.6$% for the sample. We note that this can only be measured accurately with a small step size during the ramping of the applied voltage.

The breakthrough voltage provides even more valuable insights into oxide behavior through determination of the defect density at the junction interface. If thickness inhomogeneities occur or defects are located at the oxide interface, electrical breakthrough can appear at far lower voltages (defect-related) than when there are no weak links (intrinsic). For standardized industrial processes, only a few defects occur, and for a statistical analysis, a large number of junctions need to be analyzed to distinguish between defect-related and intrinsic breakthrough. This analysis employs a probability calculation similar to that used for the breaking of chains at the weakest link. A junction can be seen again as a simple plate capacitor and subdivided in parallel paths; the overall capacitor will experience failure when the weakest capacitor in the grid breaks through. Based on the analysis of metal–oxide–semiconductor (MOS) time-dependent oxide breakthrough, the cumulative number of failed capacitors *P* up to a certain voltage serves as an indicator for the transition from defect-related to intrinsic breakthrough [23].

These studies focus on the analysis of SiO_2_ as an oxide barrier for MOS devices, whereas our approach is accessing $\mathrm{Al}/{\mathrm{Al}}_{x}O_{y}/\mathrm{Al}$ for JJ fabrication. At the transition point of the two regimes, the critical defect density $D_{crit}$ leading to the defect-related breakthrough can be derived as well as the corresponding critical electric field strength $E_{crit}$. For the analysis, the electrical field strength $E=V_{BT}/{\bar{t}}_{ox}$ can be calculated from the applied breakthrough voltage and the wafer-mean oxide thickness. However, the point of transition, i.e., when $E_{crit}$ is reached, can only be seen by plotting *E* in a Weibull graph, see Figure 6b. We find for all our data a comparable $E_{crit}=4.4$ MV/m^2^, which is in accordance with the literature [7] and further confirms the accuracy of this approach. For the assumption of random defect spread, a Poisson distribution can be used. The critical defect density $D_{crit}$ results from the probability of finding defect-free capacitors

(9) $$D_{crit}=\frac{-ln(1-P_{k})}{A},\;$$

with $P_{k}$ the probability at the transition [24]. Our samples showed for the reference TJs a critical defect density of 70 cm^−2^, which was increased for lower oxide thicknesses by etching (Table 1). The increase in $D_{crit}$ could be attributed to an increase in the oxide surface roughness with increasing etching time. However, this would imply an increase in roughness by a factor of 80, which is unrealistic for polycrystalline Al films, as the etch rates and roughness for the different Al crystal orientations are still comparable [25]. Therefore, we conclude that the increase in $D_{crit}$ should be due to an increased amount of surface traps induced by the wet etching. Nonetheless, we note that the trap densities estimated for our junctions are in all cases relatively low (up to only 5500 defects/cm^2^). Defects created during etching, particularly in the TJs, have a negative impact on the homogeneity of the interface and render the structure unsuitable for qubit fabrication. Our work shows that the process optimization and quality parameters relevant for quantum application at cryogenic temperatures can be discerned using electrical measurements at RT. Defects and other perturbations can cause increased losses and are possible sources of two-level states [26].

## 4. Conclusions

Various $\mathrm{Al}/{\mathrm{Al}}_{x}O_{y}/\mathrm{Al}$ junctions have been characterized at RT. Wafer-scale capacitance measurements, combined with EELS, enabled a reliable determination of the effective oxide thicknesses of junctions across full 200 mm wafers. From this analysis, we find that our subtractive fabrication approach yields junctions with quite homogeneous “average” oxide thicknesses across the full wafers, with a thickness spread of less than 3%. This value is particularly important for achieving precise qubit frequency targeting on full 200 mm wafers.

Moreover, the analysis of leakage currents, by taking into account different contributions to the current across the whole junction interface, enabled us to infer the oxide uniformity within each junction. From this, we confirm a very uniform control of oxide thickness attained with our wet etching approach, which is important for the scaling of the junction area. Based on detailed analysis of current–voltage characteristics, different charge transport mechanisms across the junctions could be distinguished. At low voltages the transport is ohmic, associated with direct tunneling across the oxide barrier. At higher voltages, the onset of Fowler–Nordheim tunneling is recognizable. As no intermediate regime is identified, trap-assisted tunneling processes are absent; i.e., high-quality oxides can be prepared by the combination of plasma processing and wet-chemical etching. From the direct tunneling regime, we could calculate the tunnel coefficient *k* for all oxide thicknesses. We found that *k* increases with decreasing oxide thickness, pointing to a stoichiometry gradient across the oxide barrier width.

Additionally, we could show that detailed statistical analysis of the breakthrough voltages of junctions can provide valuable wafer-level information about interface defect densities. For our junctions, we calculated defect densities in the range 70–5500 defects/cm^2^. The density increases with decreasing oxide thickness (increasing etching time), indicating that wet etching is involved in the formation of defects. The ability to identify defects in the junction interface with electrical RT measurements is of utmost importance for optimizing TJs for superconducting qubits. Our study demonstrates the value of advanced RT characterization in extracting tunnel junction parameters and their spatial distribution across full 200 mm wafers, which are essential for process monitoring and optimization in industrial superconducting qubit manufacturing. 

## Figures and Tables

**Table 1:** Parameter values derived for all samples from the RT electrical characterization data.

Sample	Etch (s)	*C*/*A* (fF/µm^2^)	${\bar{t}}_{\mathrm{ox}}$ (nm)	*k* (nm^−1^)	*RA* (MΩµm^2^)	*RA_S_* (MΩµm^2^)	${\bar{V}}_{\mathrm{BT}}$ (V)	$E_{\mathrm{crit}}$ (MV/cm)	$D_{\mathrm{crit}}$ (cm^−2^)
reference	–	20.07 ± 0.03	4.4	15.7	(10.9 ± 0.6)$\cdot 10^{3}$	(8.9 ± 0.8)$\cdot 10^{3}$	2.18 ± 0.06	4.5	70
etch10	10	25.99 ± 0.06	3.5	17.8	13.5 ± 0.6	5.9 ± 0.1	1.66 ± 0.07	4.4	1700
etch20	20	27.08 ± 0.06	3.3	18.4	2.9 ± 0.1	2.17 ± 0.04	1.56 ± 0.07	4.3	2100
etch30	30	28.9 ± 0.1	3.1	19.3	1.50 ± 0.03	1.24 ± 0.02	1.51 ± 0.07	4.6	5500

## Data Availability

The original contributions presented in this study are included in the article. Further inquiries can be directed to the corresponding authors.
